# Applying Mathematical Optimization Methods to an ACT-R Instance-Based Learning Model

**DOI:** 10.1371/journal.pone.0158832

**Published:** 2016-07-07

**Authors:** Nadia Said, Michael Engelhart, Christian Kirches, Stefan Körkel, Daniel V. Holt

**Affiliations:** 1 Institute of Psychology, Heidelberg University, Hauptstr. 47–51, 69117 Heidelberg, Germany; 2 Interdisciplinary Center for Scientific Computing (IWR), Heidelberg University, Im Neuenheimer Feld 205, 69120 Heidelberg, Germany; University of Groningen, NETHERLANDS

## Abstract

Computational models of cognition provide an interface to connect advanced mathematical tools and methods to empirically supported theories of behavior in psychology, cognitive science, and neuroscience. In this article, we consider a computational model of instance-based learning, implemented in the ACT-R cognitive architecture. We propose an approach for obtaining mathematical reformulations of such cognitive models that improve their computational tractability. For the well-established *Sugar Factory* dynamic decision making task, we conduct a simulation study to analyze central model parameters. We show how mathematical optimization techniques can be applied to efficiently identify optimal parameter values with respect to different optimization goals. Beyond these methodological contributions, our analysis reveals the sensitivity of this particular task with respect to initial settings and yields new insights into how average human performance deviates from potential optimal performance. We conclude by discussing possible extensions of our approach as well as future steps towards applying more powerful derivative-based optimization methods.

## Introduction

Modern cognitive architectures, such as ACT-R [1], allow researchers to construct computational models of behavior that adequately reflect the complexity of human cognition while still being fully formalized. Cognitive architectures are typically based on empirical behavioral studies and neurophysiological research. Using a cognitive model of decision making, it becomes possible to answer questions such as “how does a typical decision maker behave in a particular situation” or “what can be expected, in the best or worst case, from a decision maker”.

Cognitive *models* usually focus on specific cognitive phenomena, while cognitive *architectures* are concerned with the general structure of the cognitive system across different tasks. Different types of cognitive architectures based on symbolic, connectionist, or hybrid frameworks exist, such as Soar [2, 3], Leabra [4], Nengo [5], and ACT-R [1]. The increasing availability and use of formal models in the behavioral sciences provides a foundation for applying advanced mathematical tools and methods [6, 7].

**Parameter Identification** The behavior exhibited by a cognitive model typically depends on multiple *model parameters*, e.g., the rate of memory decay or the amount of cognitive noise. Understanding the parameter space of a given cognitive model and efficiently estimating parameter values that best match an expected or measured behavior is a central task in cognitive modeling. This task is made difficult by the large number of function evaluations required, and by the necessary computational complexity of relevant models. Exploring the effects of different parameter values in a cognitive model is important to fully understand its behavior, to identify parameter combinations providing the best fit to human data, and to analyze sensitivity towards parameter variations [8]. In practice, for cognitive models this is often still conducted manually, guided by a researcher’s intuition or simply by trial-and-error.

Developing techniques for efficient parameter space exploration and parameter estimation is still a relatively new research area in cognitive modeling, and only a few systematic approaches have been described in the literature to date, e.g. [9–13]. Systematic exploration of a model’s parameter space is often desirable, but quickly runs into difficulties, as processing time increases exponentially with the number of parameters and the resolution of analysis (*curse of dimensionality*). While parallel high-performance computing can improve the speed of parameter space searches to some extent, this combinatorial explosion easily exceeds the capacity even of large computing resources [10].

Another possibility is to improve the efficiency of search algorithms. One approach is to sample the search space selectively, for example using adaptive mesh refinement or regression trees [9, 13], where regions of the search space with high-information content are sampled more densely. This strategy allows to preserve most of the information relevant for modeling purposes, while reducing the number of samples required.

Instead of attempting to approximate the full parameter space, it is sometimes sufficient to identify particular points or areas with certain characteristics, e.g., parameter combinations that provide the best model fit to empirical data. To reach this goal, *heuristic optimization methods* such as genetic algorithms have been employed, which use an evolutionary generate-and-select-strategy to find optimal parameter combinations [11, 12]. These heuristic approaches, however, not only require drastically higher computational resources with increasing number of dimensions, but also usually do not come with a proof of optimality of the obtained parameter estimate. Using *mathematical optimization methods*, these issues may partially be avoided by taking information found in (approximations of) first order derivatives of model and objective function into account. This, however, requires an appropriate mathematical reformulation of the model.

**Contribution** This article proposes an optimization-based approach for evaluating the behavior of a cognitive model of instance-based learning implemented in the ACT-R cognitive architecture. We propose to rewrite the model in terms of mathematically tractable expressions and to apply methods from mathematical programming in order to identify parameter values that are optimal with respect to a prescribed criterion. Our approach is generic in the sense that it may be applied to any ACT-R model based on declarative working memory, and may in principle be automated. Extensions to a much wider class of ACT-R models are possible.

To illustrate our approach, we work with an ACT-R model of the *Sugar Factory* dynamic decision making task [14–16]. We first conduct a simulation study for the analysis of two central model parameters. We then show how to address two common optimization problems: Firstly, the identification of parameter values for the best model fit to human reference values, and, secondly, the determination of parameter values that maximize the performance score. In addition to heuristic optimization methods, we apply derivative-based methods that, given an initial guess, construct descent paths to a minimizer instead of searching the entire parameter space, thereby improving computational efficiency.

Beyond these methodological contributions, our analysis allows us to quantify to what extent performance in the *Sugar Factory* task depends on initial conditions, which is informative for the experimental use of the *Sugar Factory*. Our results furthermore yield new insights into how average human performance deviates from potential optimal performance.

## Mathematical Optimization

Our aim is the application of mathematical optimization methods to a cognitive model of decision making to optimize its fit to human behavioral data and to identify conditions of optimal performance. To this end, we formulate mathematical optimization problems and choose appropriate mathematical optimization methods to solve them efficiently.

**Optimization Targets** Our dynamic decision making task setting is round-based, where we denote rounds by *j* = 1, …, *N*_*r*_. For a given parameter vector *θ*, the model behavior may also depend on a pseudo-random sequence of inputs. Then, evaluations take place over repetitions *i* = 1, …, *n* with differing realizations of the pseudo-random input sequence. We consider two optimization tasks with respect to the model of the cognitive process:

1. **Fit optimization**. We determine a parameter vector $\theta \in R^{n_{\theta }}$ that gives rise to best model fit to human reference values. For optimizing the model fit, the objective function is the root mean square deviation (RMSD) of the model performance and a human reference value *R*_ref_,
  $$\begin{array}{c} \underset{\theta }{\text{min}}\;\sqrt{\frac{1}{n}\sum_{i=1}^{n}{\left(R^{i}\left(\theta \right)-R_{\text{ref}}\right)}^{2}}, \end{array}$$ (1)
  where $R^{i}\left(\theta \right)=\sum_{j=1}^{N_{r}}R_{j+1}^{i}\left(\theta \right)$. Herein, $R_{j+1}^{i}\left(\theta \right)$ denotes a zero-one indicator that the process was *on target*, i.e. a certain prescribed goal was reached, in repetition *i* after round *j* and for model parameters *θ*.
2. **Performance optimization**. We determine a parameter vector $\theta \in R^{n_{\theta }}$ with best score. The objective function for the best score is a weighted sum consisting of the performance criterion, here the mean of the rounds on target, and its standard deviation,
  $$\begin{array}{c} \underset{\theta }{\text{max}}\;a\;\cdot \;\frac{1}{n}\sum_{i=1}^{n}R^{i}\left(\theta \right)+b\;\cdot \;\sqrt{\frac{1}{n-1}{\sum_{i=1}^{n}\left(R^{i}\left(\theta \right)-\frac{1}{n}\sum_{i=1}^{n}R^{i}\left(\theta \right)\right)}^{2}}. \end{array}$$
  Constants a,b∈R are weighting factors.

## Mathematical Reformulation of the ACT-R Model

**Fig 1:**
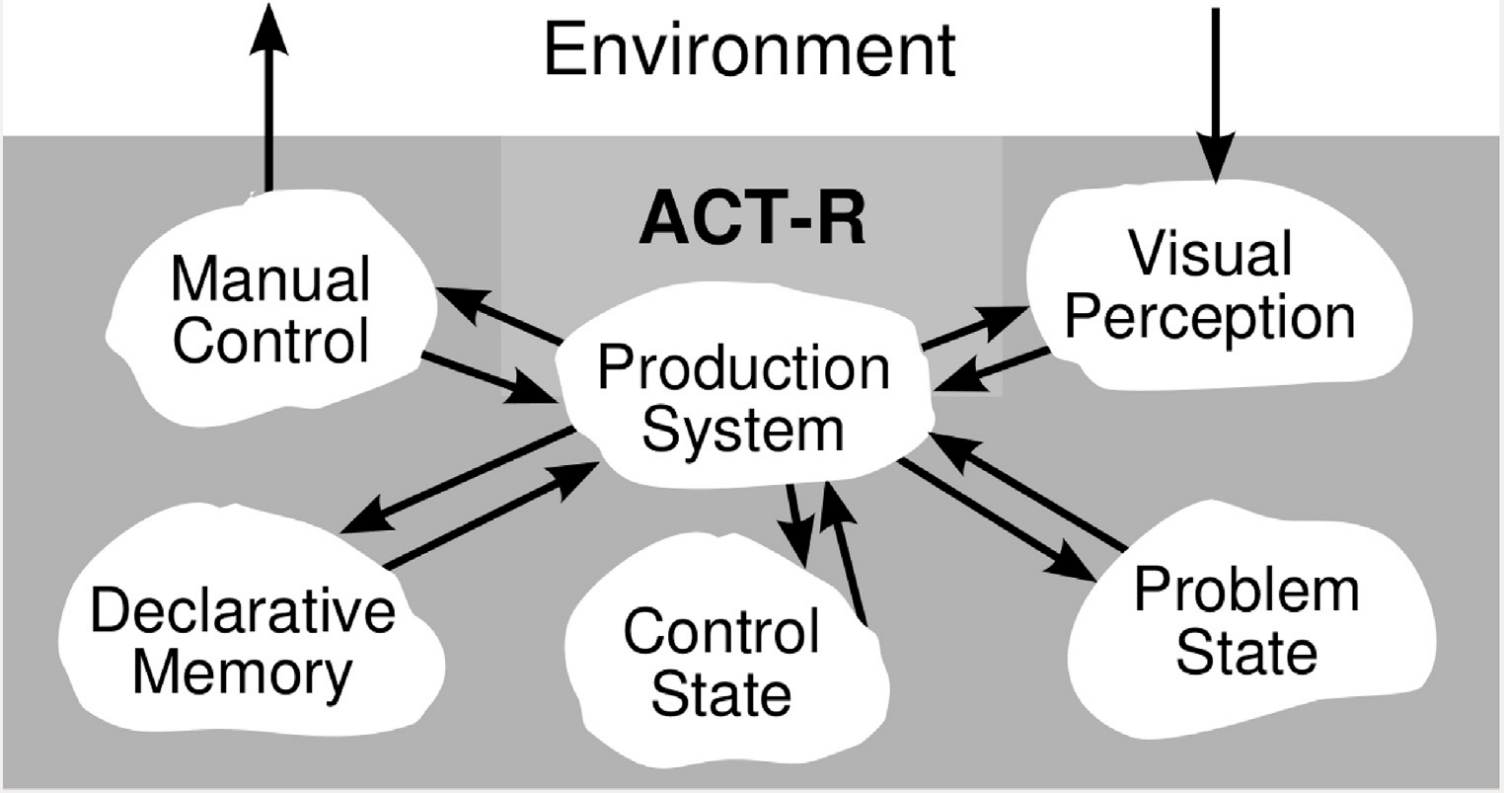
Connection of modules in ACT-R 5.0 [18].

The cognitive architecture used in this article is ACT-R, a computational framework for modeling higher level cognition [1]. ACT-R consists of three main components: modules, buffers, and a pattern matcher, which are associated with distinct cortical regions. A central production system coordinates the behavior of these modules, see Fig 1. In several functional magnetic resonance imaging (fMRI) studies, Anderson et al. (2007) [17] identified a number of brain regions corresponding to modules in ACT-R, supporting the structure of the architecture.

One important feature of ACT-R is that it combines the symbolic structure of cognition, i.e., how knowledge is encoded as high-level structures, with subsymbolic processes which represent an “[…] abstract characterization of the role of neural computation in making that knowledge available,” [17]. As an example, instances of symbolic declarative knowledge (e.g., “The number 2 is followed by the number 3.”), called chunks, are stored in declarative memory. On the subsymbolic level, an activation value is associated with each chunk and determines whether the information is accessible in a certain situation (e.g., when counting). In contrast to purely connectionist models, in which specific cognitive phenomena emerge from interconnected networks of simple units [19], ACT-R operates on different levels of abstraction in order to achieve a representation of how the components of the mind interact.

### Mathematical Description of the Declarative Memory Module

The proposed reformulation of the *Sugar Factory* task (see below) includes a generic representation of a central part of the ACT-R cognitive architecture, the *declarative memory module*. Our approach can therefore be applied in a straightforward manner to other cognitive tasks that rely on this cognitive module.

**Fig 2:**
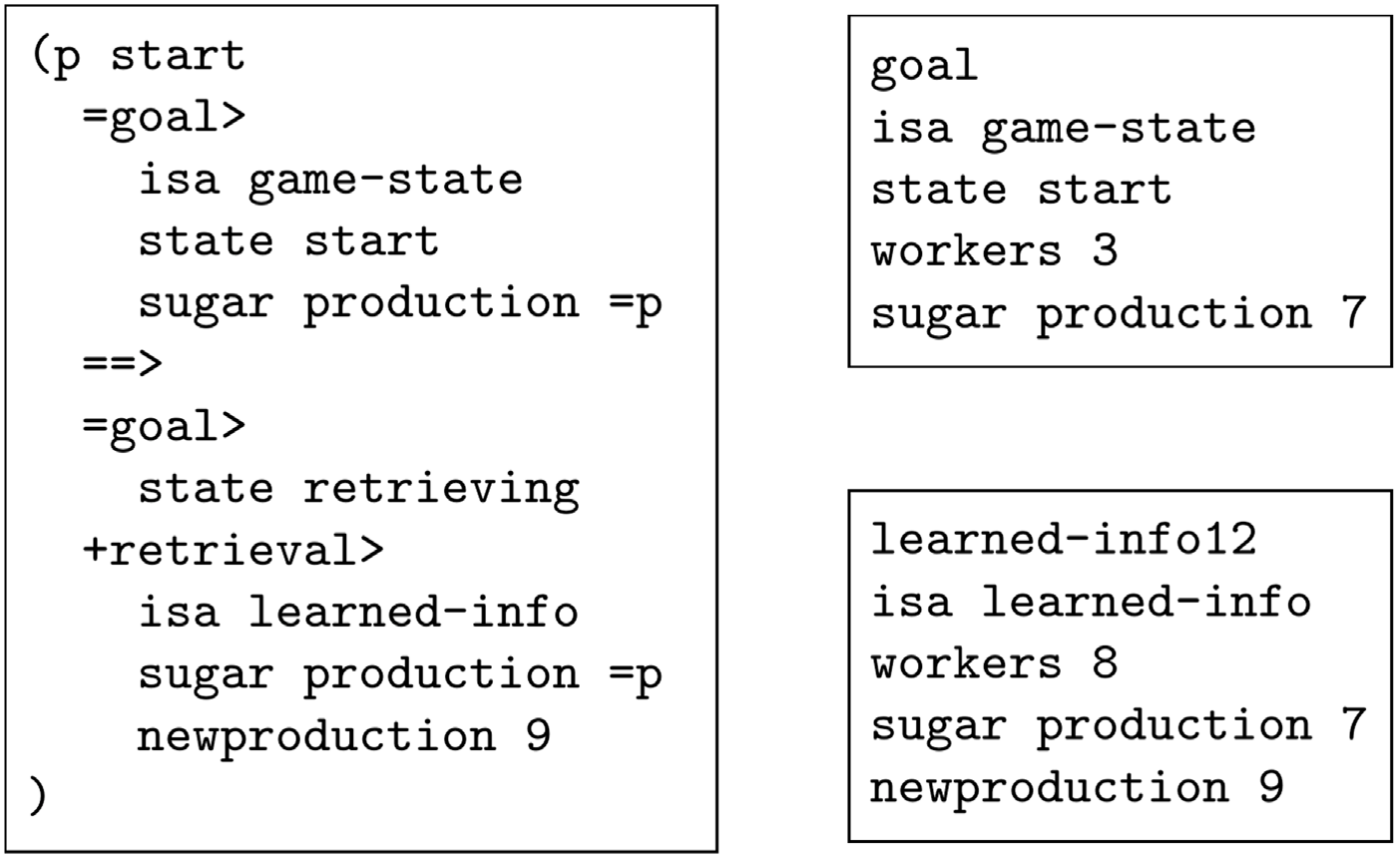
Examples of an ACT-R production rule (left) and of ACT-R chunks stored in declarative memory (right).

A single element of declarative knowledge is called a *chunk*, stored in the *declarative memory* module of the ACT-R architecture. A chunk, see Fig 2, is defined by its chunk type and contains a number of *slots* *c*_*ik*_ that hold information. Each chunk also has an *activation value* *A*_*i*_ that reflects the usefulness of the stored information for the specific situation at hand [17].

**Definition 1 (Chunk and Declarative Memory)** *A chunk is a tuple* $\left(c_{i1},\ldots ,c_{ik},A_{i}\right)\in I_{1}\times \ldots \times I_{z}\times R$. *The declarative memory is an ordered list* M *of chunk tuples indexed by consecutive ascending natural numbers*.

The current context and all past experience influence the activation value *A*_*i*_ of a chunk *i*, which is computed from three components: the base-level activation *B*_*i*_, a context component *C*_*i*_, and a noise component $u_{ij}^{\text{n}}$,

$$\begin{array}{c} A_{i}:=B_{i}+C_{i}+u_{ij}^{\text{n}}. \end{array}$$ (2)

The base-level activation *B*_*i*_ is calculated from the number *n*_*i*_ of presentations of a chunk, its lifetime *L*_*i*_, and the decay parameter *d*,

$$\begin{array}{c} B_{i}:=\ln\left(n_{i}/\left(1-d\right)\right)-d\ln\left(L_{i}\right). \end{array}$$ (3)

A chunk is said to have been *presented* when it first enters declarative memory, *n*_*i*_ = 1, or when it is recalled or encountered again, *n*_*i*_ > 1. With each additional presentation of a chunk, the base-level activation *B*_*i*_ increases [17]. The lifetime *L*_*i*_ is the time since creation of the chunk. In the case of the *Sugar Factory* implementation, *L*_*i*_ is calculated from the round *t*_*i*_ of a chunk’s creation, the current simulation round *j*, and a time constant *T* = 0.05 s,

$$\begin{array}{c} L_{i}:=\left(j-t_{i}\right)+T. \end{array}$$ (4)

When faced with the current situation in turn *j*, a *retrieval request* is be made to retrieve a chunk from declarative memory that best matches the current situation. Then, from the subset of chunks that are a satisfactory match of request (*p*_*j*_, p*), comprised of a *situation* *p*_*j*_ and a *desired target* p*, the one with the highest activation value is placed into the *retrieval buffer*.

**Definition 2 (Retrieval of a Chunk)** *Given a retrieval request* (*p*_*j*_, p*), *the index of the chunk retrieved from declarative memory is*

$$\begin{array}{c} i^{*}=\underset{i}{\text{argmax}}\left\{A_{i}\left(p_{j},p^{*}\right)\geq \tau \right\}. \end{array}$$ (5)

*The retrieval threshold* *τ* *defines the minimum activation threshold for a chunk to be retrievable at all. The retrieved chunk is*

$$\begin{array}{c} \left(c_{1}^{*},\ldots ,c_{k}^{*},A^{*}\right)=(c_{i1^{*}},\ldots ,c_{ik^{*}},A_{i^{*}}\left(p_{j},p^{*}\right)). \end{array}$$ (6)

The context component *C*_*i*_(*p*_*j*_, p*) contributes a similarity part that reflects the similarity between the slot values (*p*_*j*_, p*) of a retrieval request and the slot values (*c*_*i*1_, …, *c*_*ik*_) of any chunks in declarative memory. It is not required that the slots of the chunk have exactly the same values as specified in the retrieval request, but *C*_*i*_ increases if their similarity is high. This mechanism is called *partial matching*,

$$\begin{array}{c} C_{i}\left(p_{j},p^{*}\right):=P\cdot \sum_{l}M_{i,l}, \end{array}$$ (7)

wherein the parameter *P* reflects the amount of weighting given to the similarities, and the similarity measures *M*_*i*, *l*_ are calculated as

$$\begin{array}{c} M_{i,l}\left(a,b\right):=-\left|a-b\right|/\max\left(a,b\right). \end{array}$$ (8)

Maximum similarity between two values is represented by *M*_*i*, *l*_ := 0 and maximum dissimilarity by *M*_*i*, *l*_ := −1.

Finally, the noise value $u_{ij}^{\text{n}}$ added to the activation consists of two subcomponents: a transient component $u_{ij}^{\text{n}}$, which is computed each time a retrieval request is made, and a permanent component, which is only generated once for each chunk. The transient component is usually sufficient for modeling. To generate the value of the transient noise component a logistic distribution with *μ* = 0 and noise parameter *s* ≈ 0.2 is used [20].

### Mathematical Description of the Task and Production Rules

Different modules of ACT-R interact with each other through a production system. The steps the *Sugar Factory* model runs through are described below. In every round:

1. compute the activations of memory chunks;
2. select the chunk with the highest activation regarding a particular recall request;
3. if there is such a chunk and the activation of this chunk is above the threshold *τ*: make choice stored in chunk *b*^1^;
4. if there is no such chunk or the activation of the chunk is lower than the threshold *τ*: make random choice *b*^2^;
5. update the *Sugar Factory* system state;
6. create a new memory chunk or merge information acquired with an existing chunk.

Both general cognitive processes and production rules for simulating performance in a specific task are described in ACT-R by a system involving logical relations. In contrast, we aim to formulate a continuously differentiable mathematical model that is a suitable input to mathematical optimization methods. The relevant logical phrases from the ACT-R formalism are argmax, | ⋅ | and max, and conditional *if-then* statements. We propose formulations for all three components based on the Heaviside and Delta functions *H*(*x*) and *δ*(*x*):

$$\begin{array}{c} H\left(x\right)=\left\{\begin{array}{cc} 1 & \text{if}\;x\geq 0,\\ 0 & \text{if}\;x<0, \end{array}\right.,\;\delta \left(x\right)=\left\{\begin{array}{cc} 1 & \text{if}\;x=0,\\ 0 & \text{if}\;x\neq 0. \end{array}\right. \end{array}$$ (9)

**Formulation of *if-then* statements**. We write *if-then* statements

$$\begin{array}{c} x\left(s\right)=\left\{\begin{array}{cc} a, & \text{if}\;s\geq 0,\\ b, & \text{if}\;s<0, \end{array}\right.\;y\left(t\right)=\left\{\begin{array}{cc} c & \text{if}\;t=0,\\ d & \text{if}\;t\neq 0. \end{array}\right. \end{array}$$

as *x*(*s*) = *H*(*s*) ⋅ *a* + (1 − *H*(*s*)) ⋅ *b* and *y*(*t*) = *δ*(*t*) ⋅ *c* + (1 − *δ*(*t*)) ⋅ *d*.

**Formulation of** max **and** | ⋅ |. We substitute max and | ⋅ | by

$$\begin{array}{cc} \max(x,y) & =H(x-y)\cdot x+(1-H(x-y))\cdot y,\\ \frac{\left|x-y\right|}{\max(x,y)} & =H\left(x-y\right)\frac{x-y}{x}+\left(1-H\left(x-y\right)\right)\frac{y-x}{y}. \end{array}$$

**Formulation of argmax**. To evaluate the statement

$$\begin{array}{c} i^{*}=\underset{1\leq i\leq n}{\text{argmax}}\left\{A_{i}\right\},\;x_{j}\left(i^{*}\right)=\left\{\begin{array}{cc} b^{1} & \text{if}\;A_{i^{*}}\geq \tau ,\\ b^{2} & \text{if}\;A_{i^{*}}<\tau , \end{array}\right. \end{array}$$

we first compute $A^{*}=\max_{i}\left\{A_{i}\right\}$, and then let

$$\begin{array}{c} x_{j}\left(i^{*}\right)=\sum_{i=1}^{n}H\left(A_{i}-A^{*}\right)\cdot \left(H\left(A^{*}-\tau \right)\cdot b^{1}+\left(1-\left(A^{*}-\tau \right)\right)\cdot b^{2}\right). \end{array}$$

In order to obtain a continuously differentiable formulation, we then replace Heaviside and Delta functions by continuous approximations,

$$\begin{array}{cc} H(x) & :=\frac{1}{\pi }\arctan\left(hx\right)+\frac{1}{2},\\ \delta (x) & :=\exp(-x^{2}/a^{2}), \end{array}$$

with, e.g., *h* = 10.0, *a* = 0.01.

### The Sugar Factory Task

In this article, we investigate an ACT-R model of the *Sugar Factory* decision making task [16]. The *Sugar Factory* is a turn-based task realized as a computer-based simulation, which was developed by [14] to study how people learn to control intransparent dynamic systems. Instead of inducing explicit rules for controlling the system, participants seem to store instances of situation-response pairs in memory, which are retrieved when a similar situation occurs, see [15, 16]. This cognitive mechanism is known as *instance-based learning* (IBL), cf. [21], and has been shown to play an important role in dynamic decision making, e.g., [22]. IBL has been implemented successfully in several cognitive models based on the ACT-R architecture as reported in [16, 22].

In the *Sugar Factory* task, participants control a simulated sugar factory. They are asked to reach and maintain a specific level of sugar production p* over turns *j* = 1, …, *N*_*r*_ by repeatedly changing the number of workers *x*_*j*_ employed at the factory. The initial production is *p*_1_ = 6. In every round *j*, the goal is to reach a production of *p*_*j*_ = p* = 9, i.e., to produce 9,000 metric tons of sugar. The following equation describes the behavior of the *Sugar Factory* task.

**Definition 3 (Sugar Factory Simulation Problem)** *The sugar production rate before turn* *j* = 1 *is* *p*_1_ *and the rate* *p*_*j*+1_ *after turn* *j* = 1, …, *N*_*r*_ *is given by*

$$\begin{array}{c} p_{j+1}\left(x\right)={\left(2\cdot x_{j}-p_{j}\left(x\right)+u_{j}^{\text{r}}\right)}_{\left[1,12\right]}, \end{array}$$ (10)

*where* *x*_*j*_ ∈ {1, …, 12} *is a sequence of inputs*, $u_{j}^{\text{r}}$ *is uniformly distributed random variable from* {−1, 0, 1}, *and* (*y*)_[*a*, *b*]_ = max(*a*, min(*b*, *y*)) *denotes the clipping of the argument value* *y* *to the range* [*a*, *b*].

Participants are initially unaware of the relationship Eq (10) between workers and sugar production, and are not informed about their results being evaluated in this way.

To measure the performance of a participant in the *Sugar Factory*, we define the following score.

**Definition 4 (Sugar Factory Score Function)** *The sugar factory score function is*

$$\begin{array}{c} R=\sum_{j=1}^{N_{r}}R_{j+1}=\sum_{j=1}^{N_{r}}\chi \left\{|p_{j+1}\left(x\right)-p^{*}|\leq 1\right\} \end{array}$$

*with* p* = 9, *i.e., the score counts the number of rounds where the sugar production rate is on target*.

To account for the randomness in $u_{j}^{\text{r}}$ and to make it possible for participants to be on target 100% of the time, a sugar production of *p*_*j*_ ∈ [8, 10] is also scored as being on target.

### Human Performance in the Sugar Factory

It has repeatedly been found that human participants are able to control the simulated system above chance level but perform far from the theoretical optimum in this task [14, 15]. Moreover, even successful participants are often unable to verbally describe the structure of the system. This is in line with the assumptions of instance-based learning as a cognitive mechanism which does not require the abstraction of formal rules or relations. Surprisingly, even when the structure of the underlying system is made explicit to participants, they are generally not able to improve their performance [14].

Analyzing individual decision behavior, [15] found that up to 86% of the initial ten choices *x*_1_, …, *x*_10_ made by participants can be explained by the following rules, which form the basis for the cognitive model further below:

- Initially, a workforce of *x*_1_ = 7, 8, or 9 is chosen;
- If the sugar production is below or above target, *p*_*j*_ < 8 or *p*_*j*_ > 10, then $x_{j}=x_{j-1}+u_{j}^{\text{off}}$, where $u_{j}^{\text{off}}\in \{-2,\ldots ,2\}$ is added to the current workforce;
- If the sugar production is on target, 8 ≤ *p*_*j*_ ≤ 10, then $u_{j}^{\text{on}}\in \{-1,\ldots ,1\}$ is added to the current workforce.

**Box 1. Algorithm 1: Mathematical formulation of the ACT-R model of the *Sugar Factory*.**

**for** *j* = 1, …, *N*_*r*_ **do**

  **(1)** **for** *i* = 1, …, *N*_*c*_ **do**

     *L*_*i*_ := (*j* − *t*_*i*_) + *T*;

     *B*_*i*_ := ln (*n*_*i*_/(1 − *d*)) − *d* · ln (*L*_*i*_);

     *M*_*i*1_ := −|*p*_*j*_ − *c*_*i*2_|/max(*p*_*j*_, *c*_*i*2_);

     *M*_*i*2_ := −|9 − *c*_*i*3_|/max(9, *c*_*i*3_);

     *A*_*i*_ := *B*_*i*_ + *P* · (*M*_*i*1_ + *M*_*i*2_) + $u_{ij}^{\text{n}}$;

    **end**

  **(2)** i* := argmax_*i*_{*A*_*i*_};

  **(3)** *A*_i*_ ≥ *τ*?

     (i) if *A*_i*_ ≥ *τ* then *x*_*j*_ := *c*_i*1_;

     (ii) else      *x*_*j*_ := *u*_*w*,*j*_;

  **(4)** $p_{j+1}:=2\cdot x_{j}-p_{j}+u_{j}^{\text{r}}$;

     (i) if *p*_*j*+1_ > 12 then *p*_*j*+1_ = 12;

     (ii) if *p*_*j*+1_ < 1 then *p*_*j*+1_ = 1;

     (iii) *p*_*j*+1_ = 9?

       (a) if *p*_*j*+1_ = 9 then  $u_{w,j+1}:=u_{w,j}+u_{j}^{\text{on}};$

       (b) else       $u_{w,j+1}:=u_{w,j}+u_{j}^{\text{off}};$

  **(5)** if *u*_*w*,*j*+1_ > 12 then *u*_*w*,*j*+1_ = 12;

  **(6)** if *u*_*w*,*j*+1_ < 1 then *u*_*w*,*j*+1_ = 1;

  **(7)** *p*_*j*+1_ ∈ {8, …, 10}?

     (i) if *p*_*j*+1_ ∈ {8, …, 10} then  *R*_*j*+1_ := 1;

     (ii) else           *R*_*j*+1_ := 0;

  **(8)** ∃*i* = 1, …, *N*_*c*_: *c*_*i*_ = (*x*_*j*_, *p*_*j*_, *p*_*j*+1_)?

     (i) if ∃*i* then *n*_*i*_ := *n*_*i*_ + 1

     (ii) else    *N*_*c*_ := *N*_*c*_ + 1;

         $c_{N_{c}}:=(x_{j},p_{j},p_{j+1})$;

         $n_{N_{c}}:=1$;

         $t_{N_{c}}:=j$;

**end**

As an example and to demonstrate the mathematical description of the production rules, the limits on the sugar production rates in the *Sugar Factory* are implemented by *if-then* statements. These rules appear as follows in our mathematical description:

$$\begin{array}{c} \text{if}\;p_{j+1}>12\;\text{then}\;p_{j+1}=12,\;\text{if}\;p_{j+1}<1\;\text{then}\;p_{j+1}=1. \end{array}$$

In our reformulation, these *if-then* statements are smoothened using the Heaviside function *H*:

$$\begin{array}{lll} {\tilde{p}}_{j+1} & = & 2\cdot x_{j+1}-p_{j}+u_{rj},\\ p_{j+1} & = & H({\tilde{p}}_{j+1}-12)\cdot 12+(1-H({\tilde{p}}_{j+1}-12))\\ & & \cdot (H(1-{\tilde{p}}_{j+1})\cdot 1+(1-H(1-{\tilde{p}}_{j+1}))\cdot {\tilde{p}}_{j+1}). \end{array}$$

### Nonlinear Recurrence Model

For the *Sugar Factory* problem, let *N*_*r*_ be the number of rounds. Each chunk *i* has three slots (*c*_*i*1_, *c*_*i*2_, *c*_*i*3_), where *c*_*i*1_ holds the information about the new workforce, the value *c*_*i*2_ represents the current production and *c*_*i*3_ is the new production calculated from *c*_*i*1_ and *c*_*i*2_. The maximum number *N*_*c*_ of chunks can be calculated from the number of values *c*_*ik*_ possible for slot *k* ∈ {1, 2, 3} of chunk *i*. Feasible values for new workforce *c*_*i*1_, current production *c*_*i*2_, and new production *c*_*i*3_ are {1, …, 12} each. Thus, *N*_*c*_ = 12 · 12 · 12 = 1,728. We allocate every possible chunk and set its initial activity to a sufficiently small negative value −*M* to make sure that it is possible to activate it only after information has been stored in the slots of the chunk.

The mathematical model contains different types of variables:

- *states* including the activation *A*_*i*_ of the chunks, the current number of workers *x*_*j*_, and the current sugar production rate *p*_*j*_ in the *Sugar Factory*,
- *parameters* *θ* = (*τ*, *d*, *P*) and *s* describing selected cognitive properties of the individual participant, and
- *pseudo-random vectors*, containing the cognitive noise *u*^n^, random decisions by the participants $u_{w}+u_{j}^{\text{on}}$ resp. $u_{w}+u_{j}^{\text{off}}$ and system inputs *u*^r^. The sequences of random values are generated a priori as reproducible pseudo-random numbers.

All inputs are vectors of length *N*_*r*_, except $u_{ij}^{\text{n}}$, which is of length *N*_*r*_ ⋅ *N*_*c*_. The value *R*_*j*+1_ is used as an indicator whether the participant has reached the target in round *j*, i.e., whether the new sugar production *p*_*j*+1_ equals 8, 9, or 10. The overall score *R*^*i*^ is computed by summation over all *R*_*j*+1_,

$$\begin{array}{c} R^{i}=\sum_{j=1}^{N_{r}}R_{j+1}^{i}. \end{array}$$

with $R_{j+1}^{i}$ as the indicator *on target* in round *j* = 1, …, *N*_*r*_ for input *i* = 1, …, *n*. This modeling approach leads to a system of nonlinear recurrence relations as shown in Box 1.

## Properties of the Model and Choice of Optimization Methods

We have implemented the mathematical reformulation of the *Sugar Factory* model in *Python*. Our implementation is modular and object-oriented, with model-specific components encapsulated in a problem class that is easy to substitute in order to transfer it to similar tasks. In this section, we report simulation results obtained using this implementation.

### Simulation and Sensitivity Analysis

Computations of the simulations were run on a non-dedicated 48-core machine (4 × 12-core *AMD Opteron 6176*) with 256 GB RAM. To give an impression of the computational effort involved, the maximum runtime for searching even the finest grid investigated did not exceed one day. There were no noticeable differences between the runtimes of our *Python* and the standard ACT-R implementation.

We focused on an analysis of the parameters *P* (weighting given to similarities) and *τ* (retrieval threshold), which have considerable effect on the achieved score. The decay rate was set to its default value *d* = 0.5. The activation noise $u_{ij}^{\text{n}}$ was set to zero as it does not lead to a noticeable change of the mean score. We describe the random components *u*^on^, *u*^off^ (number of workers added to the current workforce depending on whether the sugar production is on or off target), and *u*^*r*^ (random variable added to the sugar production) by pseudo-random input sequences.

**Fig 3:**
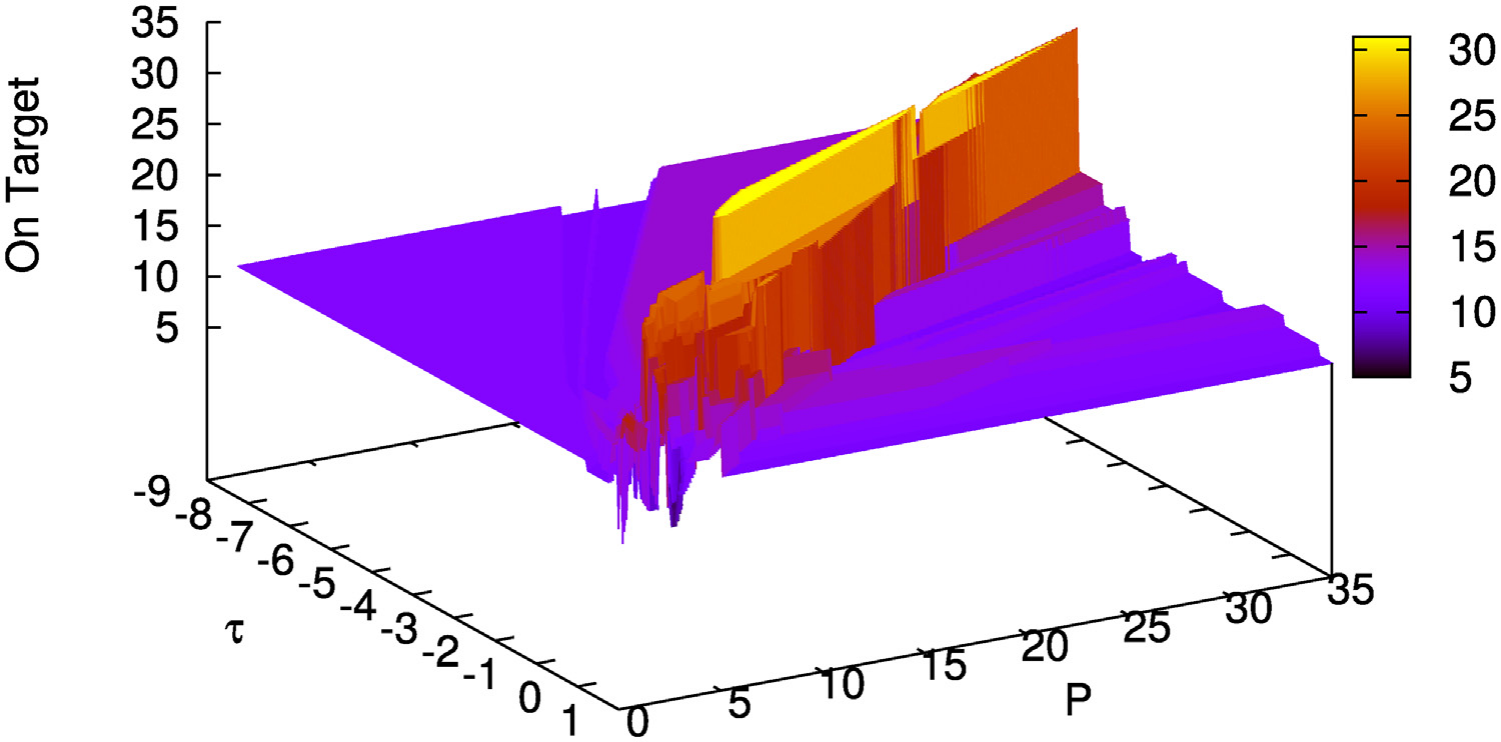
Rounds on target for input_0_ (= $u_{0}^{\text{on}}$, $u_{0}^{\text{off}}$, $u_{0}^{\text{r}}$) over 40 rounds on fine parameter grid with 66,086 grid points. With $u_{0}^{\text{on}}\in \{-1,\ldots ,1\}$, $u_{0}^{\text{off}}\in \{-2,\ldots ,2\}$, and $u_{0}^{r}\in \{-1,0,1\}$.

**Fig 4:**
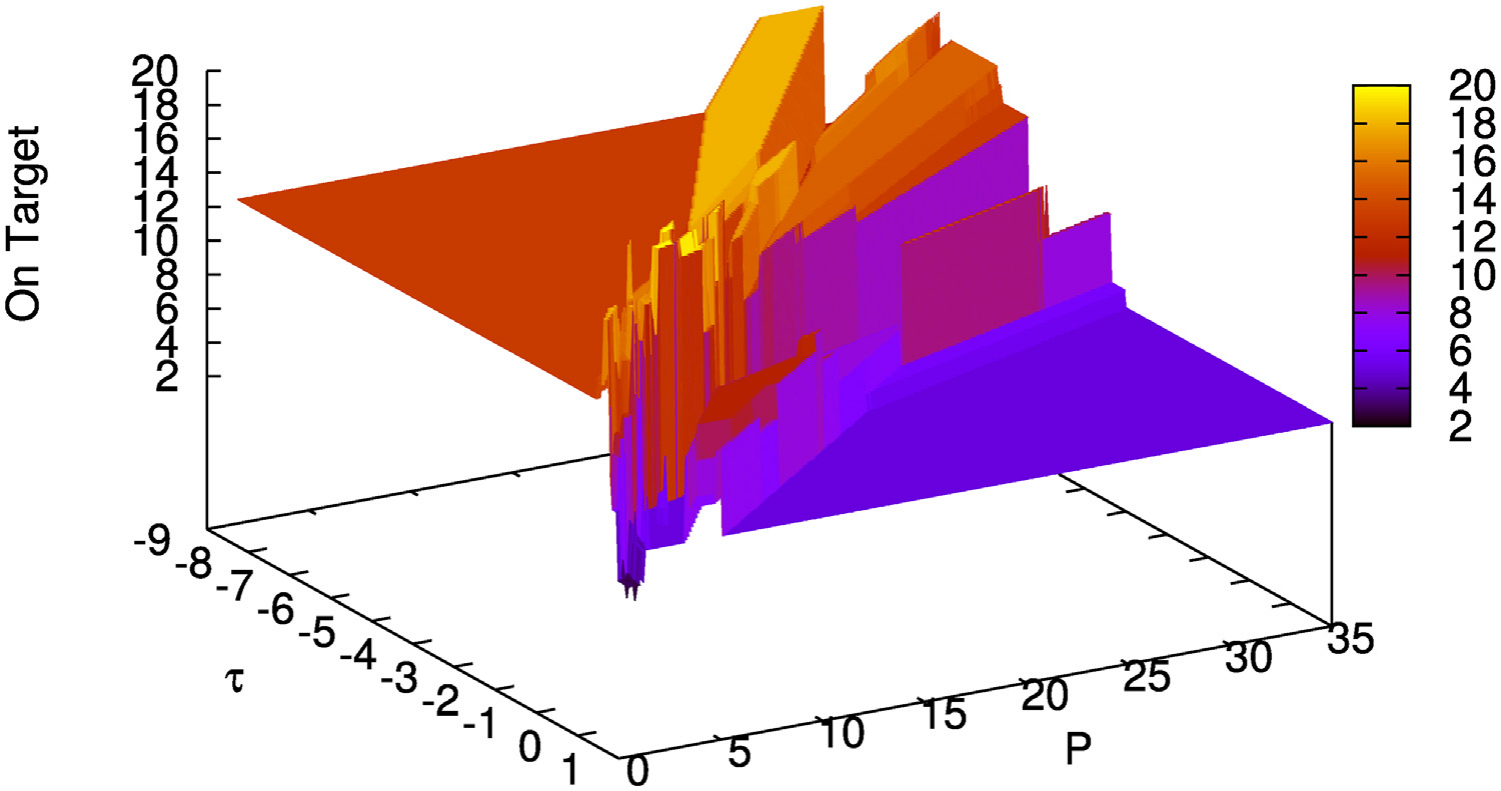
Rounds on target for input_1_ (= $u_{1}^{\text{on}}$, $u_{1}^{\text{off}}$, $u_{1}^{\text{r}}$) over 40 rounds on fine parameter grid with 66,086 grid points. With $u_{0}^{\text{on}}\in \{-1,\ldots ,1\}$, $u_{0}^{\text{off}}\in \{-2,\ldots ,2\}$, and $u_{0}^{r}\in \{-1,0,1\}$.

Fig 3 shows simulation results for one fixed input (i.e., for every parameter combination (*τ*, *P*) the same input sequences *u*^on^, *u*^off^, and *u*^*r*^ were used). Parameter ranges are *τ* ∈ [−8.50, 1.00] with step size Δ*τ* = 0.05, and *P* ∈ [0.5, 35.0] with step size Δ*P* = 0.1, which results in a total of 66,086 sampling points. There are certain parameter combinations for which the model is on target about [87.5]% of the time (*τ* ∈ [−3.3, −0.85] and *P* ∈ [8.4, 23.7]) and others where the score drops to less than 25%. The structure of the plots, especially in the area of the *best learners*, strongly depends on the pseudo-random inputs, compare Figs 3 and 4. In the latter, the *best learners* are on target no more than 50% of the time, the score drops to 10% near the edge. This difference in performance arises from the effect of early random decisions of the model (determined by the pseudo-random input sequences $u_{0}^{\text{on}}$ and $u_{0}^{\text{off}}$) as memory chunks *on target*, which are important for guiding model behavior, are created early by lucky random choices.

**Fig 5:**
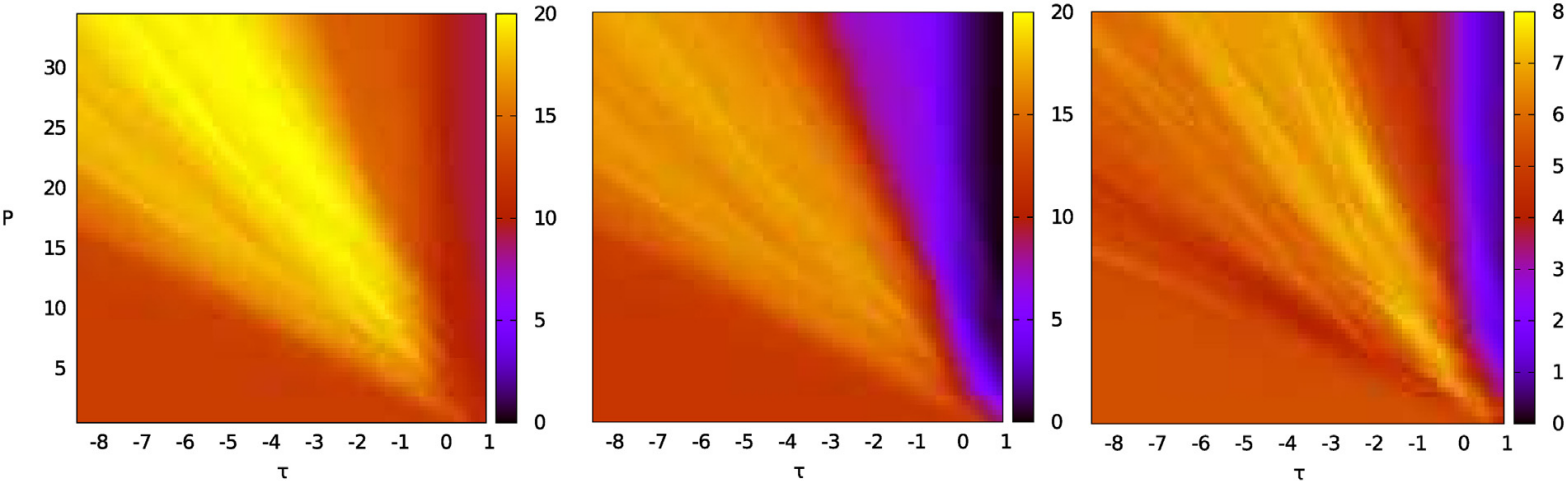
Mean value and standard deviation of rounds on target for 100 different inputs over 40 rounds. Initial sugar production rate *p*_1_ = 6, medium parameter grid with 8,256 grid points. Left: Mean value of rounds on target. Center: Mean value, activated chunks only. Right: Standard dev., activated chunks only.

Hence, we conducted a second simulation in which the input sequences were varied pseudo-randomly. Fig 5 (left) shows the mean performance for 100 different pseudo-random sequences. Not only does the total number of rounds on target differ compared to the single inputs, but the area of parameter combinations that yield good results is also much broader. To investigate to what extent performance depends on learning, only trials in which a previously learned chunk was retrieved were counted in Fig 5 (center), using the same 100 pseudo-random sequences. Compared to Fig 5 (left), there is a drop of the score in the upper right quarter of Fig 5 (center, standard deviations right). This pattern reveals that effective learning only occurred in the lower left corner (i.e., chunks are recalled) while in the upper right corner high values of *P* and *tau* impede recall and behavior is driven mostly by random choices. As detailed in the *Cognitive Interpretation* further below, loose criteria for recall (i.e., a low recall threshold and low required similarity of chunks) seem to be beneficial in this task.

In a further simulation, we investigated the sensitivity of the scores with respect to different initial sugar production values. The default value used in experiments is *p*_1_ = 6. Results for an initial value of *p*_1_ = 9 show, compared to the default value *p*_1_ = 6, a much broader region of *best solvers* and also a higher overall score. In contrast, an initial value of *p*_1_ = 1 yields a lower overall score as well as a smaller region of *best solvers*. All simulation results show a similar pattern in response to parameter variations, with the *highest scoring* parameter combinations located in a wedge-shaped area at the center of the plot and lower scores in both lower left and upper right corners. Please refer to *Cognitive Interpretation* further below for a detailed discussion of these results.

### Choice of Optimization Methods

In this section we discuss the results of our simulation study regarding the numerical differentiability of our reformulated model as well as the appropriate optimization methods. In order to apply derivative-based optimization methods, a tractable formulation is necessary. However, the ACT-R architecture contains many logical statements (e.g., *if-then-else*) and absolute value expressions that challenge a derivative-based approach. As shown before, such non-differentiabilities can be smoothed using continuous approximations of the Heaviside and Delta functions Eq (9). This is similar to the approach of Gurney et al. [23], in which the authors also used a smooth approximation of the Heaviside function in order to model action selection.

A different approach is described by Chapelle et al. [24], who used a softmax activation function and showed that it is possible to perform gradient-based optimization on their smooth approximation. This approach however requires i.a. that the index selected by argmax is unique. How to deal with chunks having (almost) the same activation remains an open question.

**Choice of the Smoothing Parameter *h*** We concentrated on the influence of the parameter *h* of the Heaviside function, as the parameter *a* of the Delta function turned out to be uncritical. Larger values of *h* correspond to sharper transitions at *x* = 0. To identify the value of *h* for which the model becomes numerically differentiable, we ran simulations for *h* ∈ {0.1, 1.0, 10, 10^2^, …, 10^7^} with *P* = 20, in the particular parameter interval *τ* ∈ [−3.16, −3.12] sampled at step size Δ*τ* = 10^−5^. We also separately varied *h* for smoothing of the similarity calculation, denoted by *h*_sim_, smoothing of argmax, denoted by *h*_argmax_, and for computation of sugar production and workers, denoted by *h*_env_.

**Fig 6:**
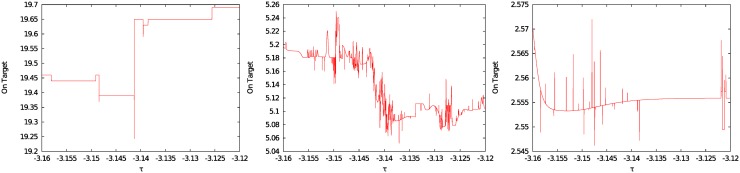
Mean value of rounds on target for 100 inputs over 40 rounds with *P* = const. Left: *h*_argmax_ = 10^7^, *h*_sim_ = *h*_env_ = 10. Center: *h*_argmax_ = 10^3^, *h*_sim_ = *h*_env_ = 10. Right: *h*_*env*_ = 10, *h*_argmax_ = *h*_sim_ = 100. Note the different vertical axis ranges.

Results in Fig 6 show that, the *argmax* term proves to be critical for matching the behavior of the *Sugar Factory* model for the ACT-R and the Python implementation. Larger values of *h* (Fig 6, left) are required but yield a numerically non-differentiable function. Decreasing the values of *h*_argmax_ leads i.a. to a random choice of chunks that undermines the learning process. Fig 6 shows that the score drops from about 19.5 (left) to approximately 5.2 (center). For the similarity calculation and the calculation of sugar production and workers, the choice of *h* is less critical, but *h*_argmax_ = 100 is still too large to yield a numerically differentiable model (right).

We may conclude that, even though smoothing the argmax can be a feasible approach, cf. [23, 24], precise modeling of the argmax is crucial at least for the particular case of the *Sugar Factory* model.

**Heuristic and Mathematical Optimization Approaches** Optimization methods such as genetic algorithms [25] or particle swarm [26] search the global parameter space based on *heuristics*. Such algorithms however rely on the computational time, for example, as termination criterion, as they have little information on whether or not they have actually found the an optimum. Two examples for such heuristic optimization methods are ESCH [27], a modified evolutionary algorithm, and Controlled Random Search (CRS) with local mutation [28]. CRS starts with a population of random points, and evolves them heuristically, a method comparable to genetic algorithms.

In contrast, *mathematical optimization solvers* are characterized by the use of derivatives as a source of additional information to make sure that an optimum is reached. Those *mathematical* optimization solvers are e.g. Newton-type algorithms, e.g. [29], or steepest descent methods, e.g. [30], but also include derivative-free methods such as Nelder-Mead [31] or BOBYQA [32], which approximate the behavior of gradient based solvers. Nelder-Mead is a downhill simplex method while BOBYQA uses an iteratively constructed quadratic approximation for the objective function. Whereas *heuristic* optimization methods are quickly stretched to their limits with an increasing dimensionality of the parameter space, the number of iterations for mathematical optimization methods, in particular for derivative based ones, ideally is independent of the problem dimensions.

### Numerical Results for the Sugar Factory

We applied a selection of heuristic and mathematical optimization algorithms that promise to cope with the non-differentiability of the nonlinear recurrence model. Our selection comprises Nelder-Mead Simplex [33] with explicit support for bound constraints [34], BOBYQA, ESCH, and CRS. All optimization algorithms were applied using the Python interface NLopt [35].

The stopping criterion for BOBYQA and Nelder-Mead was a relative tolerance on the optimization parameters of 0.1. For the heuristic global solvers ESCH and CRS we successively increased the time limit up to about 1000s. The stopping criterion was then set to the minimum run time for which there was no improvement of the found maxima observed.

**Fit optimization** Table 1 shows the results for the best fit to human reference performance, with *R*_ref_ = 7.9 taken from the literature [15]. Using multiple start values, all solvers found approximately the same point as a maximum. For ESCH and CRS the results displayed are for a time limit of 5.0 seconds.

**Table 1:** Maxima found by different solvers for *n* = 100 inputs. Objective was to find the parameter combination best fitting a human reference value using RMSD (fit optimization).

Solver	*τ*	*P*	Max.	#Eval.
Nelder-Mead	0.5	28.13	4.05	67
BOBYQA	0.5	27.80	4.05	54
ESCH	0.45	27.88	4.05	6,374
CRS	0.48	32.94	4.05	4,500

**Fig 7:**
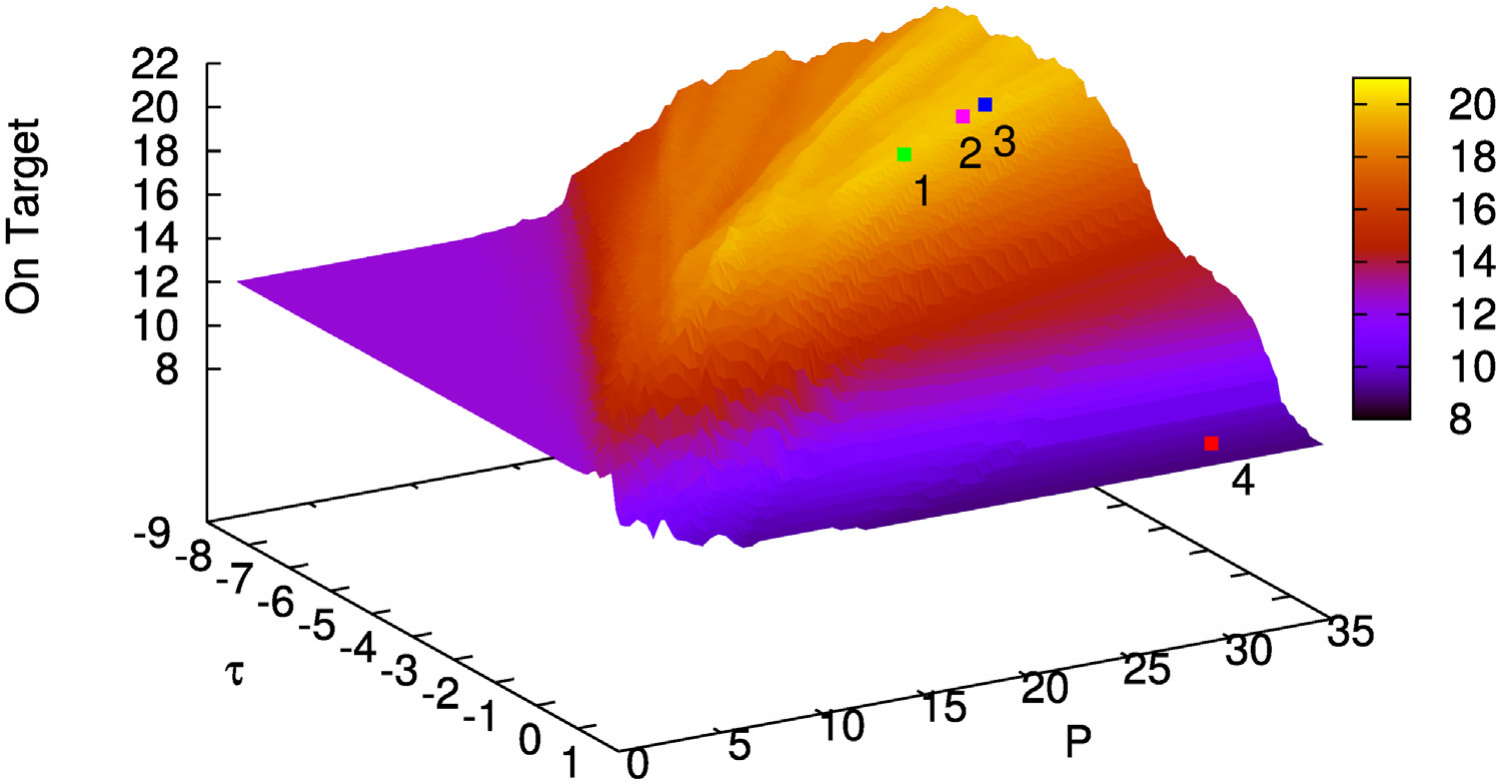
Mean of 100 inputs over 40 rounds, *p*_0_ = 6, medium grid (8,256 grid points). Points 1–4 show 1: best score found by ESCH, 2: best score found by local solvers, 3: best score found by CRS, 4: best fit to reference human.

**Performance optimization** For the single input displayed in Fig 3, all solvers found the global maximal score of 31, depending on suitable choices of the initial values for parameters *τ* and *P*. Table 2 shows the results for *a* = 1 and *b* = 0 and 100 inputs using multiple start values (see Fig 5, left). The local solvers Nelder-Mead and BOBYQA both found the same local maximum (*τ* = −4.00, *P* = 27.00 with objective = 20.15). Table 2 shows the maxima found by the heuristic global solvers after 960 seconds (see Fig 7). For *a* = 1 and *b* = −1, all solvers found the same point as a maximum (*τ* ≈ −6.5, *P* ≈ 30 with objective ≈13.87), except CRS which found a slightly better point (*τ* ≈ −8.15, *P* ≈ 34.9 with objective ≈14.04).

**Table 2:** Maxima found by different solvers for *n* = 100 inputs. Objective was to find the parameter combination with the best score (performance optimization).

Solver	*τ*	*P*	Max.	#Eval.
Nelder-Mead	-4.00	27.00	20.15	36
BOBYQA	-4.00	27.00	20.15	43
ESCH	-3.13	22.36	20.13	863
CRS	-4.21	28.52	20.2 0	860

**Fig 8:**
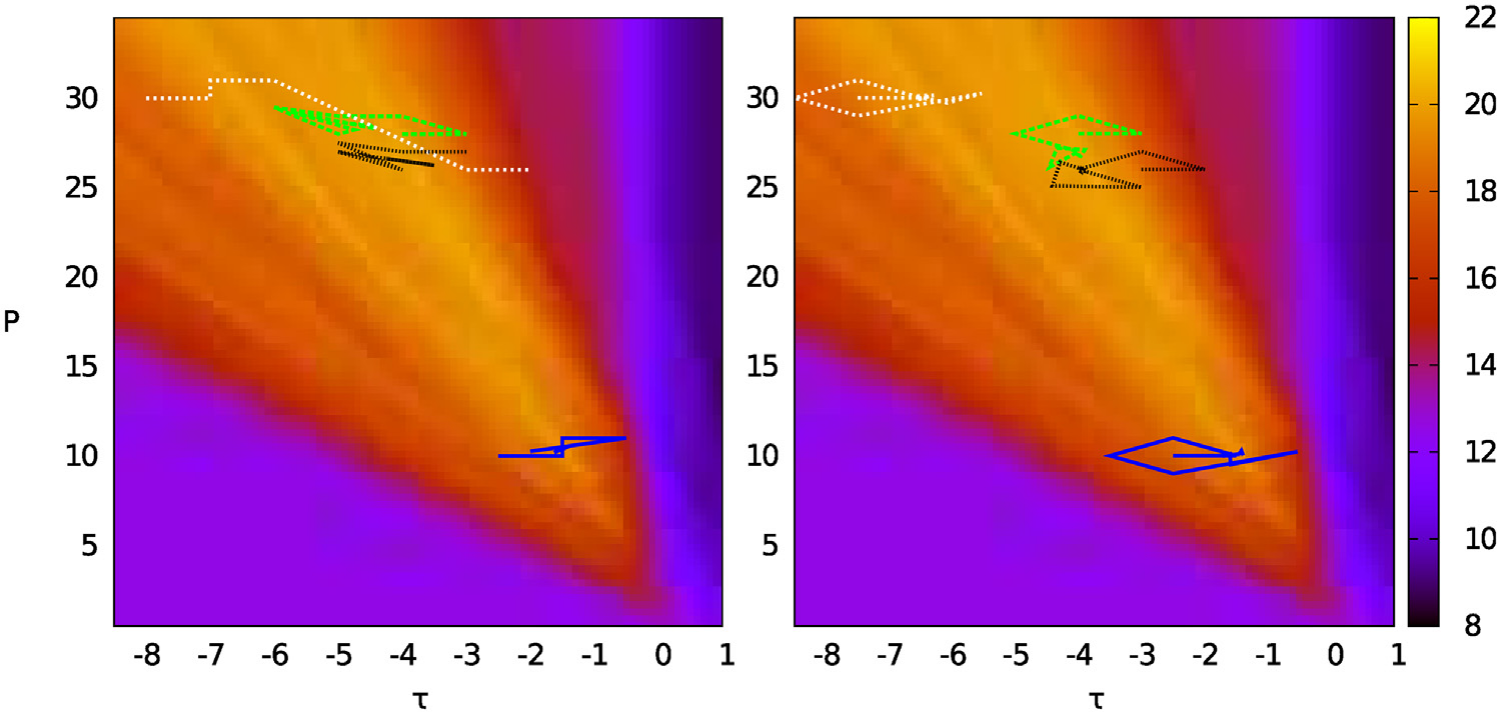
Optimization trajectories for Nelder-Mead (left) and BOBYQA (right) with four different start values.

Fig 8 shows the optimization trajectories for Nelder-Mead and BOBYQA.

### Cognitive Interpretation

Our results show how high-performance simulation and optimization can provide insights beyond just optimizing the fit to aggregate human data. Interestingly, optimizing for highest performance shows that the optimal parameter combination is considerably different from the best fit to typical human performance, particularly with respect to parameter *τ* (see Fig 7). This raises two questions, namely why the *τ* value of the model with the best fit to human performance diverges from the optimal model, and how a lower *τ* value leads to better performance (see Fig 5).

**Non-optimal human performance** A simple answer to the first question is that people do not behave formally optimal in many decision situations [36] in general, and in the *Sugar Factory* in particular [14, 15]. The structure of the human cognitive system seems to be geared towards robust information processing in typical human environments with incomplete or uncertain information [37, 38], rather than formal optimization given strict assumptions. Another possibility is that the model of human cognition used in this study is not valid to start with. However, given that existing studies of the *Sugar Factory* task and its derivatives show that implicit learning is a strong explanatory mechanism [14, 15], that the implementation of implicit learning based on the ACT-R architecture generally matches human data in other studies [39], and that the specific model used here has been empirically supported [16], we think there are good grounds to assume that the basic structure of the model is appropriate.

**Influence of the parameters *τ* and *P*** The second question is how a lower *τ* value leads to better performance (see Fig 5). Apparently, being open to considering vague memories (i.e., a low retrieval threshold *τ*) is mostly a good strategy in this task. However, this may be a task-specific effect, as participants are provided only with noise-free and correct information. Any memory, however vague, that is sufficiently similar to the target situation is therefore on average a useful source of information and likely to increase performance. If the recall threshold *τ* is high, considering chunks with a lower similarity (low similarity weighting *P*) produces a related effect. However, in contrast to *τ*, the best-fit estimate for parameter *P* lies close to the theoretical optimum for this task. The more conservative memory threshold (a high *τ* value) shown by most human participants may represent a suitable trade-off across a range of different situations, given that information often is noisy or unreliable and a higher selectivity therefore advisable. This is supported by the fact that the best-fit value of *τ* = 0.5 we found is close to the value recommended by the architecture as a suitable default for many situations (*τ* = 0) [40].

**Influence of different initial values** We also investigated how choosing different initial values for the sugar production task influenced performance. We observed that the effectiveness of instance-based learning in this task noticeably depends on the initial production values. An initial value of *p* = 9 yields the best overall performance, as it is part of the target value range, 8 ≤ *p* ≤ 10, and therefore produces memory chunks of trials *on target* early, which is important for guiding control behavior. This insight is practically relevant for behavioral studies, as the sensitivity to starting conditions has so far not been considered in studies using the *Sugar Factory*.

## Discussion

Cognitive architectures are powerful tools for modeling higher-level cognition. However, methods for efficient exploration of parameter spaces and quick parameter estimation in these models are still in the process of development. In this article, we have demonstrated first steps towards a differentiable formulation of an instance-based learning model implemented in the ACT-R cognitive architecture. We conducted a simulation study for an analysis of central model parameters and showed how mathematical optimization techniques can be applied for efficient parameter identification.

We implemented a mathematical reformulation of the *Sugar Factory*, a simple instance-based learning task, in *Python* and showed that it is possible to derive a tractable formulation. The generic part of this formulation, related to the ACT-R declarative memory module, can in principle be transferred to ACT-R models of other tasks like Backgammon [41], Air-Traffic Control [42], the beer game [43], or even more complex tasks like the Water Purification Plant [39].

Furthermore, our approach should be transferable to other ACT-R models relying on the declarative memory module. Currently we are working on a reformulation of a model of the *Digit Span Task*, which includes i.a. associative activation for modeling working memory. The approach could also be extended to cover the procedural module of ACT-R. For example, Gurney et al. (2001) [23] described a mathematical model of procedural action selection that shows several parallels to the reformulation of declarative memory presented here.

We conducted simulation studies to determine model properties by varying the parameter *h* of an approximation of the Heaviside function, which we used for smoothing the non-differentiable parts of our model. The simulations showed that in order to obtain exactly the same results like the standard ACT-R model a large *h* for the smoothened *argmax* is necessary, contrary to other parts of our model like the similarity calculation and the environmental setting (i.e. calculation of sugar production and workers). This however, leads to piecewise constant behavior of our *Python* implementation. For smaller *h* our model becomes numerically differentiable, but at the same time the learning process is replaced by random behavior. Therefore, at this stage, even though derivatives can be calculated, using gradient-based optimization methods is not feasible.

We then showed how to address two common optimization problems: Firstly, the identification of parameter values that result in a best model fit to human reference values, and, secondly, the determination of parameter values that maximize the score of a scenario. We applied both heuristic and mathematical optimization algorithms that promise to cope with the non-differentiability of our nonlinear recurrence model and showed that *mathematical optimization* solvers like Nelder-Mead Simplex or BOBYQA turned out to be the best choice for the model at hand. Not only do they have the advantage of using approximations of the derivatives to determine if an extremum is found, thus needing a lower number of iterations than the *heuristic optimization* solvers, but they are also, in principle, able to deal with higher dimensional problems. Furthermore, we conducted a simulation study for two central model parameters, the retrieval threshold *τ* and the similarity weighting *P*, using high-performance computing. Results revealed a sensitivity of the task to initial settings, a result which has not been considered in the experimental uses of the *Sugar Factory* task so far. Our findings also indicate that human performance in this specific tasks seems to be hampered in part by a tendency to be overly conservative in which memory instances are considered.

### Outlook

As the *argmax* turned out to be the crucial part of the transcribed ACT-R model, we pursued a non-differentiable approach in this article and developed a nonlinear recurrence relation that could be optimized with a selection of heuristic or derivative-free solvers. This approach has the advantage of allowing for the computation of a single round of the cognitive process by a mere function evaluation.

We envision in a next step to derive exact reformulations of IBL problems and ACT-R cognitive processes that are amenable to derivative-based optimization methods, as follows: Returning once more to the statement i* = argmax{*A*_*i*_} for data *A*_1_, …, *A*_*k*_, consider the following constrained optimization problem:

minA*,wA*s.t.A*≥Ai,1≤i≤k,wi·(Ai-A*)≥0,1≤i≤k,wi∈[0,1],∑i=1kwi=1.

Herein, A* is a free variable set to the maximum activation value by virtue of minimization and the first inequality. We seek the argmax, i.e. the index i* with *A*_i*_ = A*. All differences in the second inequality are non-positive, and all with *A*_*i*_ < A* are negative. This forces the corresponding indicators *w*_*i*_ to zero. Then, the indicator *w*_i*_ is forced to one by the equality in the third line. A function *f*(i*) depending on i*, the argmax, may then be expressed as

$$\begin{array}{c} f\left(i^{*}\right)=\sum_{i=1}^{k}w_{i}f\left(i\right), \end{array}$$

which is now bi-linear, differentiable, and independent of the argmax, but yields the same value because *w*_*i*_ = 0 for *i* ≠ i*, and *w*_i*_ = 1.

This formulation represents the computation of one sample of the dynamic decision making process by the solution of a bi-linear optimization problem. The approach is hence significantly more demanding in terms of computational effort. Moreover, optimizing over process samples computed in this way constitutes a bi-level optimization problem. Treatment of such problems is significantly more demanding also in terms of mathematical optimization algorithms, but has the advantage of precisely reproducing the sequence of chunk activations as determined by ACT-R.

Another possibility that might increase the tractability of our model is a different representation of the production rules, as in [44]. Instead of using a two-step approach like in ACT-R, production rules only have one feature, their utilities.
